# Short-term impact of COVID-19 lockdown on metabolic control of patients with well-controlled type 2 diabetes: a single-centre observational study

**DOI:** 10.1007/s00592-020-01637-y

**Published:** 2020-11-21

**Authors:** Edoardo Biancalana, Federico Parolini, Alessandro Mengozzi, Anna Solini

**Affiliations:** 1 Department of Clinical and Experimental Medicine, University of Pisa, Via Roma 67, 56126 Pisa, Italy; 2 Department of Surgical, Medical, Molecular and Critical Area Pathology, University of Pisa, Via Roma 67, 56126 Pisa, Italy

**Keywords:** COVID-19 lockdown, Metabolic control, Physical inactivity, Type 2 diabetes

## Abstract

**Aims/hypothesis:**

The strict rules applied in Italy during the recent COVID-19 pandemic, with the prohibition to attend any regular outdoor activity, are likely to influence the degree of metabolic control in patients with type 2 diabetes. We explored such putative effect immediately after the resolution of lockdown rules, in the absence of any variation of pharmacologic treatment.

**Methods:**

One-hundred and fourteen patients with adequate metabolic control took part in this single-centre, prospective, observational study. The metabolic profile tested 1 week after the end of the lockdown was compared with the last value and the mean of the last three determinations performed before the pandemic emergency (from 6 months to 2 years before).

**Results:**

After 8 weeks of lockdown, an increase of HbA1c > 0.3% (mean +0.7%) was observed in 26% of the participants; these were also characterized by a persistent elevation in serum triglycerides able to predict the worsening of glucose control.

**Conclusions:**

Lockdown determined a relevant short-term metabolic worsening in approximately one-fourth of previously well-controlled type 2 diabetic individuals; pre-lockdown triglycerides were the only parameter able to predict such derangement of glucose control.

**Electronic supplementary material:**

The online version of this article (10.1007/s00592-020-01637-y) contains supplementary material, which is available to authorized users.

## Introduction

Lifestyle is a major determinant of glucose control in type 2 diabetes, and large evidence supports the beneficial long-term effects of regular physical activity and weight loss on metabolic parameters and progression of vascular complications of the disease [1–3]. On 9 March 2020, the Italian Government established lockdown policies to fight COVID-19 disease, making a complete limitation of sports and other outdoor activities, strong changing to several daily habits, like work routine, social interactions and also diet, with a mandatory in-house meal consumption. Such compelling rules, strictly applied all over in the country for 8 weeks, deeply modified rhythms and customs of the Italian population. In a recent online survey conducted in northern Italy it was shown that nearly half of people participating in the evaluation have changed their dietary habits during confinement, eating more and, thus, gaining weight [4, 5]. Of note, people also reported an increase in anxiety level that should lead, as known, to emotional eating and increase in the assumption of the so-called comfort food which is mainly rich in simple carbohydrates [6, 7]. In such context, patients with type 2 diabetes have been more likely than non-diabetic individuals to gain weight, due to a higher basal, but a lower activity energy expenditure [8], and a potentially rapid effect of such forced change of lifestyle and recommended good clinical practice rules, like preferential outdoor life and physical exercise, would be expected in worsening metabolic control in these patients, even in those having a good and relatively stable metabolic profile. However, a recent report suggests that quarantine and lockdown might be beneficial on short-term management of type 1 diabetes [9], thus raising intriguing questions on the balance between psychological stress, physical inactivity and toning down the rhythm of own daily life.

The aim of this observational, prospective, single-centre study was to evaluate the immediate impact of the lockdown rules on the metabolic profile of a cohort of patients with type 2 diabetes and good glucose control.

## Methods

All the patients with type 2 diabetes, regularly referring (with at least two previous clinical records in the last 2 years) to the outpatient diabetes clinic of Internal Medicine section, University Hospital in Pisa, and who were previously scheduled for a follow-up visit during the lockdown for COVID-19 (9 March 2020–4 May 2020), were screened for this observational study. Exclusion criteria were: age > 85 years, first access to the outpatient clinic, type 1 diabetes. The flow chart of the study is reported in Suppl. Figure 1. In the above-reported timeframe, 242 visits were scheduled and 169 patients matching the inclusion criteria were contacted; 137 of them accepted to take part in the survey and provided information about health conditions, including body weight variations and glycaemic control (10 patients were lost due to impossible phone contact). We sent them medical prescription for routine analysis via email; 9 patients refused to do analysis due to anxiety of referring to the hospital or to a clinical laboratory, and 13 patients were not able to perform examinations because of difficulties to reach a medical laboratory close to home. One patient died for COVID-19 at the end of March, and 3 patients needed hospital admission for urgent care. Therefore, 114 patients underwent blood drawing for biochemistry within 1 week after the end of the lockdown period and transmitted us the results by a telemedicine interview.

**Fig. 1:**
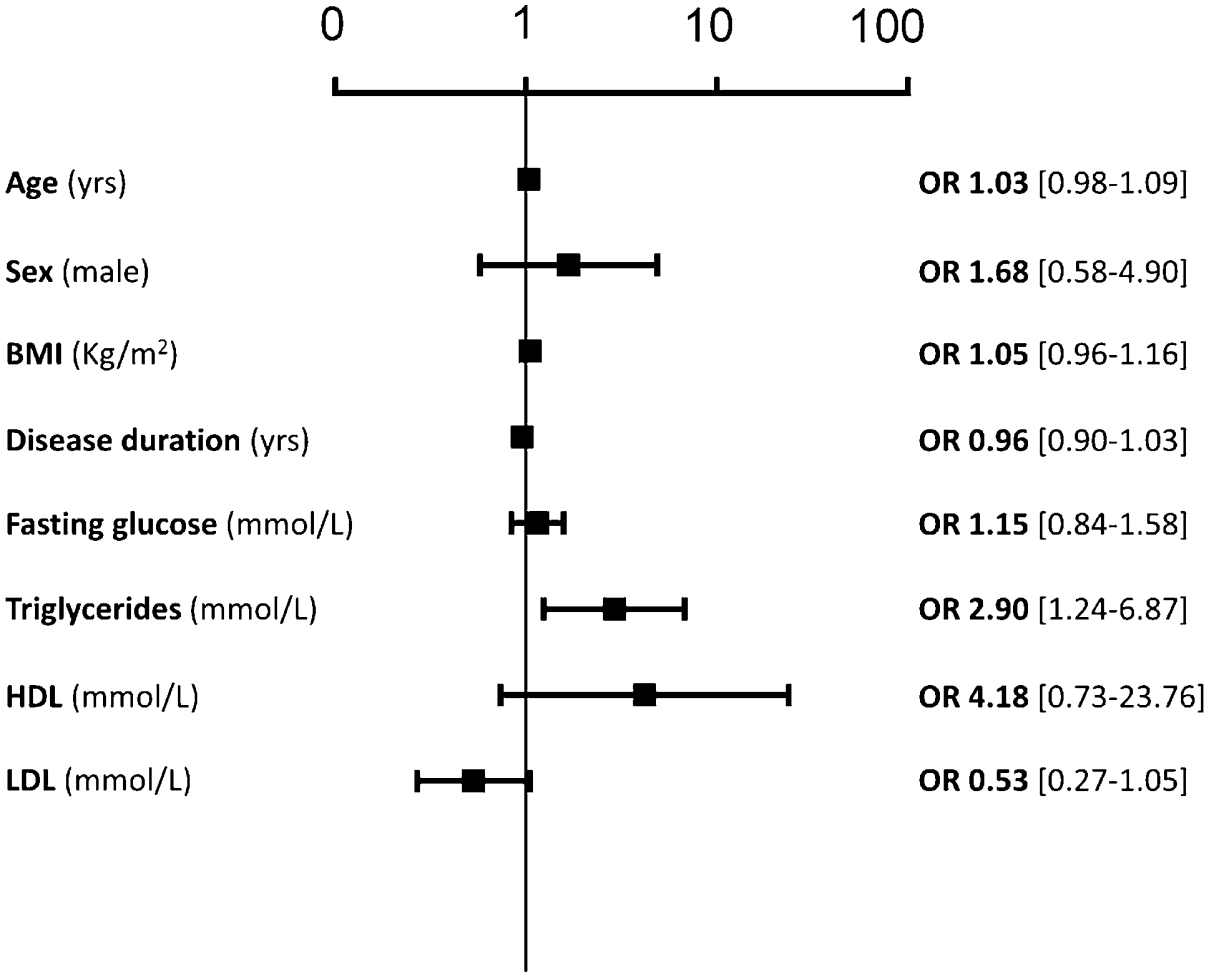
Multivariable logistic regression to calculate the risk of worse the disease for each 1-unit increase of any single variable

We then collected, for each variable of interest (fasting glucose, HbA1c, total, HDL and LDL cholesterol, triglycerides), the three last values registered in our clinical records before the pandemic time (back from 6 months up to the last two years), to make a comparison between the pre-lockdown and the post-lockdown values.

### Statistics

Data are expressed as mean (SD) or median (interquartile range) for continuous variables and number of cases (percentage) for categorical variables. Continuous variables were compared using one-way ANOVA or the nonparametric Kruskal–Wallis test. The *χ*^2^ test was applied to categorical variables. A multivariable logistic analysis was applied to calculate the risk of worsening disease for one-unit increase of each clinical variable. In order to assess differences in the fluctuation of the metabolic profile over a wide time range before lockdown, we performed a repeated-measure ANOVA taking into account the “-24 months”, “-12 months”, “pre”- (time zero) and “post-lockdown” values. A *p *< 0.05 was considered significant.

## Results

Clinical and biochemical characteristics of the whole study group are shown in Table 1. The mean age of the study cohort was 69 years with a mean duration of disease of 8.4 years (median 6.0 [2, 10]). In total, 62% of the patients were men. Patients were overweight and had preserved renal function. Looking at antidiabetic treatment, only 19.3% of the patients had insulin on board, while the rest of the cohort had oral or injective regimen; a small number were treated only with diet. In Table 2, the metabolic parameters immediately after the lockdown are compared with the last available measure before the lockdown and with the mean of the last three measures.

**Table 1:** Clinical and biochemical characteristics of the whole study cohort (*n *= 114) immediately before the lockdown

Age (years)	69.4 ± 10.3
Men (*n*; %)	71; 62.3
Disease duration (years)	8.4 ± 7.8
BMI (kg/m^2^)	28.8 ± 5.3
Insulin, with or without other agents (*n*; %)	22; 19.3
Oral or injective hypoglycaemic agents (*n*; %)	87; 76.3
Lifestyle and diet (*n*; %)	5; 4.4
Hypolipidemic agents (*n*; %)	91; 80.0
Serum creatinine (mg/dl)	0.9 ± 0.3
eGFR CKD-EPI (ml/min/1.73 m^2^)	83.7 ± 22.3

**Table 2:** Biochemical values relative to metabolic control in the whole study group (*n *= 114) before and after the lockdown (Ld)

	Last pre-Ld	Mean pre-Ld	Post-Ld	*p*
HbA1c (%; mmol/mol)	6.6 ± 0.7 48.6 ± 3.5	6.7 ± 0.7 49.7 ± 3.5	6.8 ± 0.9 50.8 ± 5.6	ns
Fasting glucose (mmol/L)	6.99 ± 1.67	7.22 ± 1.55	7.16 ± 2.11	ns
Total cholesterol (mmol/L)	4.24 ± 0.93	4.34 ± 0.88	4.13 ± 0.96	0.0210**
LDL cholesterol (mmol/L)	2.33 ± 0.83	2.35 ± 0.75	2.17 ± 0.88	0.0469* 0.0105**
Triglycerides (mmol/L)	1.50 ± 0.7	1.59 ± 0.69	1.63 ± 0.75	ns

*Mean pre*-*Ld* mean of the last three values before the lockdown

*Post-Ld versus last pre-Ld; ** post-Ld versus mean pre-Ld

Patients were divided into two groups according to the mean variation of HbA1c between the lockdown value and an average of the previous three values drawn from clinical records, defining as *Worsen* those with an increase in HbA1c > 0.3%; the remaining were defined as *Steady*. According to such stratification, 26% of the study cohort was classified as *Worsen*. Table 3 shows the phenotype and the biochemical parameters before (last measurement and mean of the last three before the lockdown); Table 4 reports deltas (Δ) of the metabolic variables. Patients with stable metabolic control tended to have lower BMI; the percent on insulin treatment did not differ with respect to the worsen ones. Regarding the metabolic profile, worsen patients displayed a significantly higher HbA1c (+0.7% vs. the last measurement before the lockdown and +0.6 vs. the last three measures, largely exceeding the target of 7% or 53 mmol/mol); also fasting glucose increased relevantly when compared to both the mean and the last value before the lockdown (Table 4). In addition, though the spontaneous changes in triglycerides were not different in the two groups, people showing a worsening of glucose control had higher triglycerides levels; interestingly, such relative difference—even with mean values within the normal range—was present already before the lockdown in these patients, while the mean values of HbA1c and fasting glucose before the lockdown were fully comparable between *Steady* and *Worsen* (Table 3). No variation in LDL cholesterol over the time was observed for both study groups.

**Table 3:** Behaviour of baseline clinical and biochemical values relative to metabolic control in patients who maintained a relatively stable glucose control over the lockdown (Steady) and in those whose glucose control worsened (Worsen)

	Steady (*n *= 85; 74%)	Worsen (*n *= 29; 26%)	*p* value
Age (years)	69.1 ± 10.4	70.5 ± 10.3	ns
Men (*n*; %)	50; 59.5	20; 69.0	ns
Disease duration (years)	8.5 ± 8.1	8.0 ± 7.2	ns
BMI (kg/m^2^)	28.4 ± 5.3	30.0 ± 5.3	ns
Insulin, with or without other agents (*n*; %)	14; 16.7	8; 27.6	ns
Oral or injective hypoglycaemic agents (*n*; %)	66; 78.5	21; 72.4	ns
Lifestyle and diet (*n*; %)	5; 4.8	0; 0.0	ns
Hypolipidemic agents (*n*; %)	66; 78.5	24; 82.7	ns
Serum creatinine (mg/dl)	1.0 ± 0.4	1.0 ± 0.2	ns
eGFR CKD-EPI (ml/min/1.73 m^2^)	79.5 ± 23.0	80.8 ± 20.0	ns
Last pre-Ld HbA1c (%; mmol/mol)	6.6 ± 0.7 48.6 ± 3.5	6.7 ± 0.8 49.7 ± 4.0	ns
Mean pre-Ld HbA1c (%; mmol/mol)	6.8 ± 0.7 50.8 ± 3.5	6.7 ± 0.8 49.7 ± 4.0	ns
Last pre-Ld fasting glucose (mmol/L)	6.83 ± 1.67	7.38 ± 1.61	ns
Mean pre-Ld fasting glucose (mmol/L)	7.16 ± 1.50	7.44 ± 1.67	ns
Last pre-Ld total cholesterol (mmol/L)	4.32 ± 0.93	4.06 ± 0.98	ns
Mean pre-Ld total cholesterol (mmol/L)	4.37 ± 0.90	4.24 ± 0.83	ns
Last pre-Ld LDL cholesterol (mmol/L)	2.40 ± 0.80	2.04 ± 0.80	ns
Mean pre-Ld LDL cholesterol (mmol/L)	2.40 ± 0.52	2.20 ± 0.70	ns
Last pre-Ld triglycerides (mmol/L)	1.43 ± 0.68	1.73 ± 0.62	0.0458
Mean pre-Ld triglycerides (mmol/L)	1.51 ± 0.61	1.86 ± 0.77	0.0138

*Ld* lockdown; *Mean pre*-*Ld* mean of the last three values before the lockdown

**Table 4:** Variations between lockdown value and last or mean values before the lockdown

	All (*n* = 114)	Steady (*n *= 85; 74%)	Worsen (*n *= 29; 26%)	*p* value
Δ mean HbA1c (%)	0.03 [− 0.3; 0.3]	− 0.075 [− 0.046; 0.06]	0.6 [0.4; 0.9]	< .0001
Δ last HbA1c (%)	0.1 [− 0.2; 0.4]	0 [− 0.2; 0.2]	0.7 [0.3; 0.9]	< .0001
Δ mean fasting glucose (mmol/L)	− 0.16 [− 0.78; 0.56]	− 0.31 [− 0.89; 0.33]	0.68 [− 0.17; 1.67]	0.0003
Δ last fasting glucose (mmol/L)	0.17 [− 0.61; 1.05]	0 [− 0.8; 0.72]	0.83 [0.15; 1.79	0.0027
Δ mean total cholesterol (mmol/L)	− 0.08 [− 0.61; 0.80]	− 0.08 [− 0.54; 0.26]	− 0.14 [0.78; 0.46]	ns
Δ last total cholesterol (mmol/L)	0 [− 0.39; 0.32]	− 0.04 [− 0.48; 0.15]	0.26 [− 0.08; 0.56]	0.0414
Δ mean LDL cholesterol (mmol/L)	− 0.1 [− 0.6; 0.34]	− 0.13 [− 0.6; 0.26]	− 0.03 [− 0.65; 0.39]	ns
Δ last LDL cholesterol (mmol/L)	− 0.03 [− 0.58; 0.32]	− 0.09 [− 0.78; 0.24]	0.17 [− 0.27; 0.46]	ns
Δ mean triglycerides (mmol/L)	− 0.01 [− 0.28; 0.25]	− 0.01 [− 0.33; 0.25]	0.03 [− 0.24; 0.2]	ns
Δ last triglycerides (mmol/L)	0.09 [− 0.29; 0.35]	0.1 [− 0.29; 0.35]	0.03 [− 0.26; 0.5]	ns

To test whether the observed metabolic fluctuations would be due to the lockdown itself, rather than reflecting an already present trend, we built up a “time curve” of metabolic control and compared the post-lockdown values of each patient with those of 12 and 24 months before the lockdown. Data are reported in Suppl Table 1. It clearly emerges from the table that the deterioration of the glucose homeostasis occurred specifically in the lockdown period, confirming the accurate separation between the two groups (*Steady* vs. *Worsen*) in terms of glucose control. Moreover, it also remarks that the difference in triglycerides was stable and persistent since at least 12 months before the lockdown.

The other metabolic variables remaining stable after the lockdown did not display major changes over the retrospective analysis.

T2DM individuals who worsened during the lockdown did not show, in the 2 years preceding pandemic, a worse metabolic control versus steady subjects, neither any particular fluctuation of their parameters of glucose control; in other words, both HbA1c and fasting glucose were relatively stable and not dissimilar from those of steady individuals. This suggests a main role of lockdown per se in determining the observed metabolic worsening.

Mean pre-lockdown triglycerides are significantly correlated with post-lockdown HbA1c (*p *= 0.0011). When we applied a multivariable logistic regression to calculate the risk of worse the disease for each 1-unit increase of any single variable, such analysis confirmed triglycerides as the only pre-lockdown parameter able to predict the worse outcome observed immediately after the lockdown. Data are shown in Fig. 1.

## Discussion

During the last months the whole scientific community put any effort in gaining knowledge in the field of COVID-19-related morbidity and mortality, as well as short-term consequences of this severe infection. In Italy, the government adopted a very strict program of lockdown, in the attempt of controlling the pandemic; such strategy deeply impacted everyone’s daily life and drove relevant medical consequences and effects also in individuals not affected by the disease, for example by determining a delay in periodic controls of outpatients carrying chronic diseases or cancer [10]. We provide here one among the first reports on the effects of the lockdown in Caucasian type 2 diabetes patients not affected by COVID-19. Our observations can be summarized as follows: (1) the lockdown induced a relevant short-term metabolic worsening in approximately one-fourth of previously well-controlled type 2 diabetic individuals; (2) such deranged glucose is mirrored by both HbA1c and fasting glucose; (3) in the 2 years preceding the pandemic, glucose control was superimposable in worsen and steady patients; (4) pre-lockdown triglycerides are the only parameter able to predict such worsening.

Noteworthy, restrictions required by COVID-19 pandemic determined a fast significant variation of HbA1c in a relevant percent of individuals, indicating a short-term impact of lifestyle changes also in this cohort with on target HbA1c. Even more, HbA1c values in the 2 years preceding the pandemic were similar in worsen and steady patients, suggesting a main role of lockdown per se in determining the observed metabolic worsening. We obviously did not have the chance to check body weight because clinic visits were forbidden, and all data provided here come from a telemedicine approach with web transmission of biochemical parameters. However, the majority of the patients raised concern about their probable weight gain during the lockdown; matter of fact, worsen patients had a tendentially higher pre-lockdown BMI, major determinant of impaired glucose control and comorbidities in type 2 diabetes [11, 12]. In this view, a concomitant role for reduced physical activity and more sedentary lifestyle cannot be excluded, considering the detrimental metabolic impact of even a short-term inactivity [13].

The impact of lockdown on patients who worsen glucose control was clinically relevant, with a +0.7% (+ 3.5 mmol/mol) for HbA1c and + 20 mg/dL (+1.1 mmol/L) for fasting glucose. None of the participants, as from telemedicine interview, reduced or changed their medications during the lockdown. The combined raise in serum triglycerides, not ample but still significant, points out the role of a variation in dietary habits and reinforces the need for not toning down the attention on an adequate lifestyle program, with regular physical activity and a correct dietary approach, even when a successful pharmacologic treatment is ongoing [14, 15].

Interestingly, the only pre-lockdown parameter able to predict what happened in these patients was fasting triglycerides, slightly but significantly higher in patients who worsened glucose control; such higher level had been recorded also 1-year pre-lockdown in this cohort. Recently, a large observation showed that high-carbohydrate diet tended to raise fasting triglycerides levels more that high-fat one [16], being also related to higher mortality [17]. Even though we did not ask the patients specifically on the grain and flour derivates consumption, we might speculate a role for an increased use of these products, given that a + 150% of flour and groats consumption has been reported by social media in our country in that times.

Triglycerides are strongly related to non-esterified fatty acids and their noxious effect on glucose homeostasis due to mechanisms of lipotoxicity [18]; even more, they represent an independent cardiovascular and renal risk factor [19, 20]. Our findings move in line with the current literature supporting the concept that triglycerides in the range of low normality are recommended [19, 21].

In conclusion, a short-term variation of daily habits induced by the COVID-19 lockdown in Italy (one of the very few countries in the world to apply a true and complete lockdown for months) significantly worsened metabolic control in a relevant number of patients with well-controlled type 2 diabetes periodically attending a tertiary diabetes clinic; high triglycerides, even within the normal range, might predict and anticipate such deleterious effect.

## Electronic supplementary material

Below is the link to the electronic supplementary material.

Supplementary material 1 (DOCX 97 kb)

Supplementary material 2 (DOCX 15 kb)

## Data Availability

Data supporting the results presented in the paper are available upon reasonable requests.

## References

[1] American Diabetes Association (2020). Facilitating behaviour change and well-being to improve health outcomes: standards of medical care in diabetes-2020. Diabetes Care.

[2] Franz MJ, Boucher JL, Rutten-Ramos S, VanWormer JJ (2015). Lifestyle weight-loss intervention outcomes in overweight and obese adults with type 2 diabetes: a systematic review and meta-analysis of randomized clinical trials. J Acad Nutr Diet.

[3] Apovian CM, Okemah J, O’Neil PM (2019). Body weight considerations in the management of type 2 diabetes. Adv Ther.

[4] Scarmozzino F, Visioli F (2020). Covid-19 and the subsequent lockdown modified dietary habits of almost half the population in an Italian sample. Foods.

[5] Zachary Z, Brianna F, Brianna L (2020). Self-quarantine and weight gain related risk factors during the COVID-19 pandemic. Obes Res Clin Pract.

[6] Stanton R, To QG, Khalesi S (2020). Depression, anxiety and stress during COVID-19: associations with changes in physical activity, sleep, tobacco and alcohol use in Australian adults. Int J Environ Res Public Health.

[7] Ghosh A, Arora B, Gupta R, Anoop S, Misra A (2020). Effects of nationwide lockdown during COVID-19 epidemic on lifestyle and other medical issues of patients with type 2 diabetes in north India. Diabetes Metab Syndr.

[8] Caron N, Peyrot N, Caderby T, Verkindt C, Dalleau G (2016). Energy expenditure in people with diabetes mellitus: a review. Front Nutr.

[9] Bonora BM, Boscari F, Avogaro A, Bruttomesso D, Fadini GP (2020). Glycaemic control among people with type 1 diabetes during lockdown for the SARS-CoV-2 outbreak in Italy. Diabetes Ther.

[10] Sud A, Torr B, Jones ME (2020). Effect of delays in the 2-week-wait cancer referral pathway during the COVID-19 pandemic on cancer survival in the UK: a modelling study. Lancet Oncol.

[11] Ryan DH, Yockey SR (2017). Weight loss and improvement in comorbidity: differences at 5%, 10%, 15%, and over. Curr Obes Rep.

[12] Mannucci E, Monami M, Dicembrini I, Piselli A, Porta M (2014). Achieving HbA1c targets in clinical trials and in the real world: a systematic review and meta-analysis. J Endocrinol Investig.

[13] Bowden Davies KA, Pickles S, Sprung VS (2019). Reduced physical activity in young and older adults: metabolic and musculoskeletal implications. Ther Adv Endocrinol Metab.

[14] Wing RR, Look AHEAD Research Group (2010). Long-term effects of a lifestyle intervention on weight and cardiovascular risk factors in individuals with type 2 diabetes mellitus: four-year results of the Look AHEAD trial. Arch Intern Med.

[15] Doshmangir P, Jahangiry L, Farhangi MA, Doshmangir L, Faraji L (2018). The effectiveness of theory- and model-based lifestyle interventions on HbA1c among patients with type 2 diabetes: a systematic review and meta-analysis. Public Health.

[16] Jung CH, Choi KM (2017). Impact of high-carbohydrate diet on metabolic parameters in patients with type 2 diabetes. Nutrients.

[17] Dehghan M, Mente A, Zhang X (2017). Associations of fats and carbohydrate intake with cardiovascular disease and mortality in 18 countries from five continents (PURE): a prospective cohort study. Lancet.

[18] Seghieri M, Tricò D, Natali A (2017). The impact of triglycerides on glucose tolerance: lipotoxicity revisited. Diabetes Metab.

[19] Marston NA, Giugliano RP, Im K (2019). Association between triglyceride lowering and reduction of cardiovascular risk across multiple lipid-lowering therapeutic classes: a systematic review and meta-regression analysis of randomized controlled trials. Circulation.

[20] Penno G, Solini A, Zoppini G (2015). Hypertriglyceridemia is independently associated with renal, but not retinal complications in subjects with type 2 diabetes: a cross-sectional analysis of the renal insufficiency and cardiovascular events (RIACE) Italian multicenter study. PLoS ONE.

[21] Thomsen M, Varbo A, Tybjærg-Hansen A, Nordestgaard BG (2014). Low nonfasting triglycerides and reduced all-cause mortality: a mendelian randomization study. Clin Chem.

