# Supplementary figures and images for: Short-term impact of COVID-19 lockdown on metabolic control of patients with well-controlled type 2 diabetes: a single-centre observational study
Source: Acta Diabetol. 2020 Nov 21;58(4):431–6. doi: 10.1007/s00592-020-01637-y (PMC7680070; doi:10.1007/s00592-020-01637-y)

Suppl Figure 1 Flow-chart of the study.


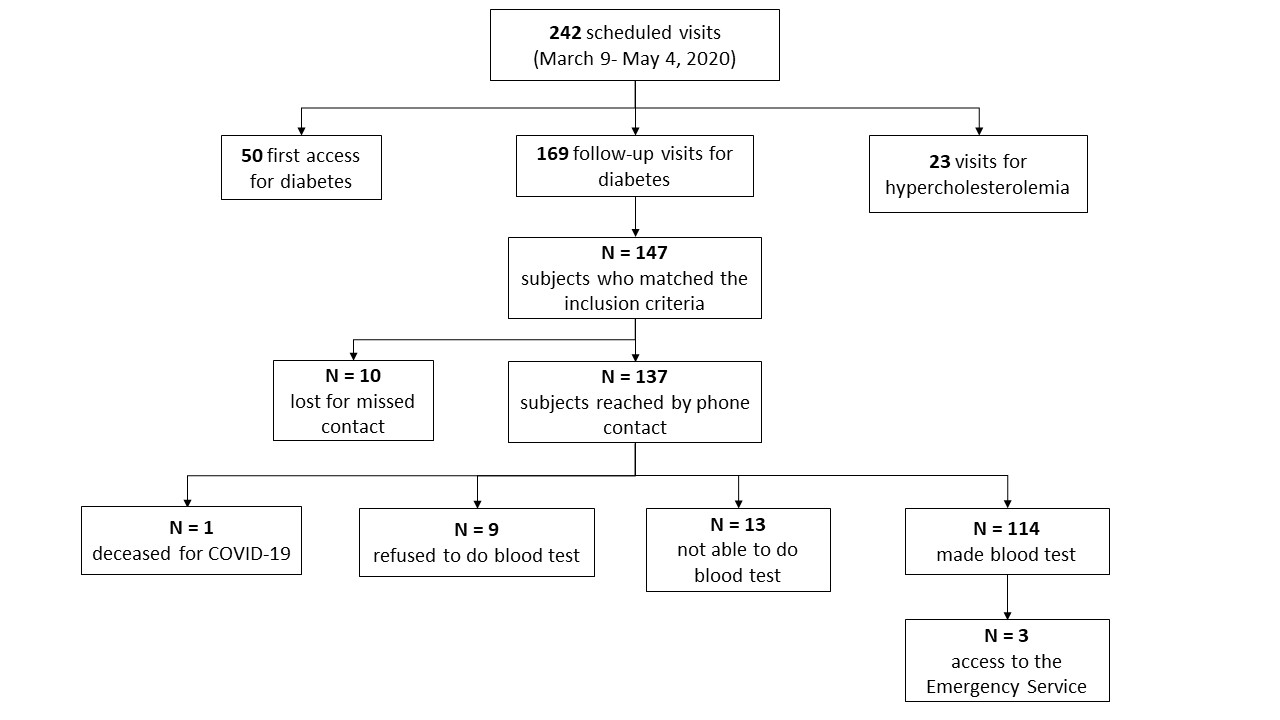

Supplement: Supplementary file 1 — Supplementary material 1 (DOCX 97 kb) [file 592_2020_1637_MOESM1_ESM.docx]
