# Supplementary material for: Short-term impact of COVID-19 lockdown on metabolic control of patients with well-controlled type 2 diabetes: a single-centre observational study
Source: Acta Diabetol. 2020 Nov 21;58(4):431–6. doi: 10.1007/s00592-020-01637-y (PMC7680070; doi:10.1007/s00592-020-01637-y)
Supplement: Supplementary file 2 — Supplementary material 2 (DOCX 15 kb) [file 592_2020_1637_MOESM2_ESM.docx]

|  | **Steady** | | | | **Worsen** | | | |  |  |
| --- | --- | --- | --- | --- | --- | --- | --- | --- | --- | --- |
|  | **Post-Ld** | **Last**  **pre-Ld** | **12 m**  **pre-Ld** | **24 m**  **pre-Ld** | **Post-Ld** | **Last**  **pre-Ld** | **12 m**  **pre-Ld** | **24 m**  **pre-Ld** | ***p value***  ***(curve)*** | ***p value***  ***(point by point)*** |
| **HbA1c**  (mmol/mol) | 47.9 ± 7.6 | 48.6 ± 3.5 | 51.1 ± 10.1 | 51.5 ± 9.4 | 59.1 ± 11.4 | 49.7 ± 4.0 | 50.8 ± 9.4 | 51.0 ± 10.0 | *<0.0001* | *< 0.0001* |
| **Fasting glucose**  (mmol/L) | 6.7 ± 1.7 | 6.8 ± 1.7 | 7.0 ± 1.6 | 7.6 ± 2.0 | 8.5 ±2.6 | 7.4 ± 1.6 | 7.5 ± 1.7 | 7.3 ± 1.7 | *0.0099* | *< 0.0001* |
| **Total cholesterol** (mmol/L) | 4.1 ± 1.0 | 4.3 ± 0.9 | 4.2 ± 0.9 | 4.4 ± 1.0 | 4.0 ± 0.8 | 4.1 ± 1.0 | 4.3 ± 0.8 | 4.4 ± 1.0 | ns | ns |
| **LDL-cholesterol**  (mmol/L) | 2.2 ± 0.9 | 2.4 ± 0.8 | 2.3 ± 0.8 | 2.4 ± 0.8 | 2.0 ± 0.7 | 2.0 ± 0.8 | 2.2 ± 0.8 | 2.4 ± 0.8 | ns | ns |
| **Triglycerides**  (mmol/L) | 1.5 ± 0.6* | 1.4 ± 0.7° | 1.5 ± 0.7§ | 1.6 ± 0.7 | 1.9 ± 1.0* | 1.7 ± 0.6° | 1.9 ± 1.0§ | 1.6 ± 1.0 | ns | **0.0093*  *°0.0458*  *§0.0114* |

**Suppl Table 1**. Comparison of the main metabolic variables over time in Steady and Worsen patients

m= months
